# Habitat transformation reshapes protistan community composition and assembly processes in coastal wetlands of southeastern China

**DOI:** 10.1128/aem.01661-25

**Published:** 2025-10-09

**Authors:** Anqi Wang, Ping Yang, Guiping Ye, Chuan Tong, Luyuan Sun, Mengmeng Feng, Zi-Yang He, Ji-Zheng He, Yongxin Lin

**Affiliations:** 1 Fujian Provincial Key Laboratory for Subtropical Resources and Environment, Fujian Normal University 12425, Fuzhou, China; 2 Research Centre of Wetlands in Subtropical Region, Fujian Normal University 12425, Fuzhou, China; 3 Fujian Key Laboratory on Conservation and Sustainable Utilization of Marine Biodiversity, Fuzhou Institute of Oceanography, College of Geography and Oceanography, Minjiang University 26465, Fuzhou, China; 4 School of Agriculture, Food and Ecosystem Sciences, Faculty of Science, The University of Melbourne 2281, Melbourne, Victoria, Australia; University of Nebraska-Lincoln, Lincoln, Nebraska, USA

**Keywords:** protists, habitat change, aquaculture reclamation, plant invasion, coastal wetlands, biogeography

## Abstract

Coastal wetlands along southeastern China have undergone extensive habitat transformations, notably from natural mudflats (MFs) to *Spartina alterniflora* marshes (SAs) and aquaculture ponds (APs), yet the ecological consequences for sediment protistan communities remain largely unknown. Here, we systematically examined protistan diversity, community composition, functional groups, and assembly processes across 21 wetlands spanning five provinces using amplicon sequencing. Although alpha diversity remained stable across habitat types, community composition and functional group distributions were significantly altered. In particular, phototrophic protists declined markedly following *S. alterniflora* invasion and then increased after aquaculture pond reclamation. Meanwhile, consumer and phototroph assemblages were strongly influenced by sediment grain size. Salinity emerged as the key environmental driver of protistan diversity and community structure. Distance-decay relationships indicated elevated spatial turnover in SAs, suggesting increased environmental filtering after plant invasion. Assembly process analysis revealed a dominance of deterministic mechanisms in shaping community structure across all habitats, with the strongest signal observed in SAs. These findings demonstrate that land-use change reshapes protistan communities through altered environmental constraints, highlighting the ecological sensitivity of microbial eukaryotes to anthropogenic disturbance in coastal wetland ecosystems.

**IMPORTANCE:**

Protists play essential roles in nutrient cycling, energy transfer, and microbial food web dynamics, yet their responses to anthropogenic habitat transformation in coastal wetlands remain underexplored. This study offers the first large-scale biogeographic assessment of sediment protistan communities across three contrasting coastal habitat types in southeastern China. We show that while alpha diversity remains resilient, profound shifts in community composition, functional group structure, and spatial turnover occur following *Spartina alterniflora* invasion and aquaculture conversion. Our findings underscore the primacy of environmental filtering, driven by salinity and sediment texture, in mediating these patterns and shaping community assembly. These insights not only expand our understanding of protistan ecology under coastal land-use change but also highlight their potential as sensitive bioindicators for monitoring ecological integrity and resilience in dynamic coastal systems.

## INTRODUCTION

Protists, defined as all eukaryotic organisms excluding plants, fungi, and animals (1), are highly diverse and abundant microbial eukaryotes in terrestrial and aquatic ecosystems. In soils and sediments, protists play pivotal roles in biogeochemical cycling, particularly by regulating microbial turnover, influencing nutrient availability, and mediating trophic interactions (2–5). Functionally, they encompass diverse trophic strategies, including consumers, phototrophs, and parasites (6, 7). In terrestrial ecosystems, consumers constitute the predominant functional group, typically outnumbering phototrophic and parasitic assemblages on a global scale (8). As keystone components of soil food webs, consumers selectively prey on bacteria, fungi, and other microbial eukaryotes, thereby enhancing nutrient mineralization, shaping microbial community composition, influencing antimicrobial resistance dynamics, and indirectly promoting plant growth and health (9–11). Through these top-down controls on microbial networks, consumers can restructure trophic interactions, thereby modulating overall ecosystem multifunctionality. Phototrophs contribute to carbon sequestration via photosynthesis (12), and may also elevate oxygen concentrations in the soil microenvironment, thus influencing redox-sensitive processes (2, 13). Parasites modulate the population dynamics and fitness of diverse hosts across multiple kingdoms, potentially altering community stability and ecosystem functioning (9). Beyond their trophic roles, protists can influence soil aggregation, organic matter decomposition, and plant-microbe interactions, thereby contributing to key ecosystem services such as carbon storage and nutrient retention (14). Owing to their short generation times, broad ecological niches, and high sensitivity to environmental fluctuations, protists have also been proposed as effective bioindicators of soil and sediment health (15). However, despite increasing recognition of their ecological roles (8), the mechanisms underlying the spatial distribution and functional differentiation of protists in complex terrestrial habitats remain poorly understood (3), particularly in comparison with bacteria and fungi (16).

Coastal wetlands represent ecotones between terrestrial and marine systems and serve as critical hotspots for nutrient transformation, sediment stabilization, and biodiversity conservation (17, 18). However, these ecosystems are under escalating pressure from global climate change and human disturbances, such as biological invasions and land-use conversions (19, 20). These perturbations profoundly reshape the physicochemical properties of wetland sediments (21, 22), alter microbial community composition (23–25), and disturb ecosystem functioning. Protists have shown marked changes in response to such disturbances: for instance, the dominance of native vs invasive plants alters protistan diversity and community structure (26), and aquaculture practices influence protistan composition depending on farming intensity and duration (27, 28).

In the past several decades, the alien grass species *Spartina alterniflora* has aggressively invaded large areas of intertidal mudflats along China’s coastline (29). In response, some invaded marshes have been reclaimed for intensive aquaculture to both curb the spread of *S. alterniflora* and meet increasing food demand (30, 31). These habitat transitions, from native mudflats to *S. alterniflora* marshes and then to aquaculture ponds, profoundly alter sediment properties, including organic matter dynamics (31–33), nitrogen transformations (34), and microbial communities such as comammox *Nitrospira* (35). Yet, despite the key ecological position of protists in structuring sediment microbial communities and mediating ecosystem processes, their responses to such sequential habitat changes remain largely unexamined.

Understanding protistan biogeography under habitat transformation requires tools that capture spatial ecological dynamics. The distance-decay relationship (DDR), where community similarity declines with increasing geographic distance, provides a quantitative framework to describe microbial spatial patterns (36–38). DDRs are shaped by both deterministic processes (e.g., environmental filtering, niche selection) and stochastic processes (e.g., dispersal limitation, ecological drift) (39, 40). The balance of these processes can differ across ecosystems and is influenced by factors such as habitat heterogeneity, hydrological connectivity, and anthropogenic disturbance (16, 41). In addition to spatial constraints, numerous abiotic factors (42), including soil moisture (43), pH (44), texture (45), salinity (46), and C/N ratio (47), as well as biotic interactions (48) and climatic conditions (7), have been shown to structure protistan communities. Assessing the relative contributions of these variables can help disentangle the mechanisms underlying community changes. While soil protistan biogeography has been explored in upland ecosystems, such as forests (48), grasslands (49), and rice paddies (5), little is known about their spatial patterns, assembly processes, and key environmental drivers in saline, tidally influenced coastal wetlands. In particular, the relative influence of plant invasion vs land-use conversion on protistan diversity and community assembly, and environmental associations remains poorly resolved.

The invasion of *S. alterniflora* and the reclamation of coastal wetlands for aquaculture represent two contrasting drivers of environmental change, one driven by biological encroachment, and the other by anthropogenic management (50, 51). *S. alterniflora* can increase sediment carbon and nitrogen storage while homogenizing bacterial communities (24), whereas aquaculture reclamation elevates sulfate levels and reduces microbial diversity (25). Given the environmental sensitivity of protists, we hypothesize that both *S. alterniflora* invasion and aquaculture reclamation will significantly reshape protistan biodiversity, community structure, and spatial turnover in coastal wetland sediments. Furthermore, we predict that aquaculture ponds, due to their stronger environmental filtering and anthropogenic control, will exert a more pronounced effect than *S. alterniflora* marshes.

## RESULTS

### Sediment physicochemical properties in different habitat types

Habitat changes significantly affected the sediment physicochemical properties in coastal wetlands (Table S1). *S. alterniflora* invasion increased soil organic carbon (SOC), NH_4_^+^-N, NO_3_^−^-N, and microbial biomass nitrogen (MBN) contents (*P* < 0.001). In contrast, aquaculture pond reclamation reduced SOC (*F*(2, 186) = 16.051, *P* < 0.001), NH_4_^+^-N (*F*(2, 186) = 23.111, *P* < 0.001), and NO_3_^−^-N content (*F*(2, 186) = 12.838, *P* < 0.001), while increasing SO_4_^2−^ content (*F*(2, 186) = 19.121, *P* < 0.001). Other environmental variables, including pH, Cl^−^, salinity, grain size (proportions of clay, silt, and sand), C/N, and microbial biomass carbon (MBC) did not differ significantly among the three habitat types.

### Diversity and community structure of protists in different habitat types

**Fig 1:**
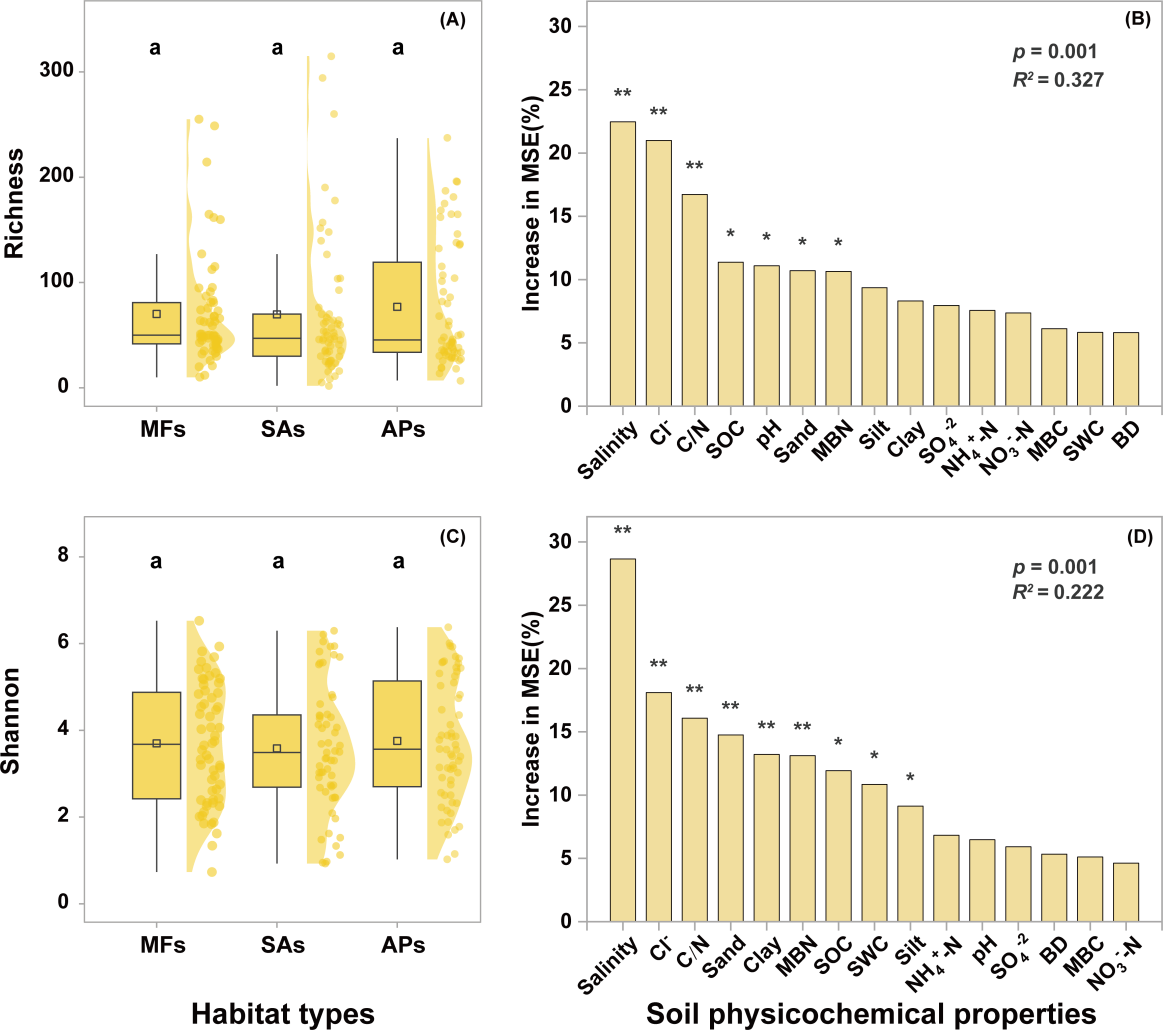
Box-normal plots showing the distribution of the richness (**A**) and Shannon diversity (**C**) of protists in the three wetland habitat types. Random forest analysis showing the importance of environmental factors in influencing the richness (**B**) and Shannon diversity (**D**) of protists. Different lowercase letters above the boxes indicate significant differences between wetland habitat types (*P* < 0.05). Asterisks indicate levels of significance (*P < 0.05; **P < 0.01; ***P < 0.001). BD, bulk density; MBC, microbial biomass carbon; MBN, microbial biomass nitrogen; SOC, soil organic carbon; SWC, sediment water content. MFs, SAs, and APs represent mudflats, *S. alterniflora* marshes, and aquaculture ponds, respectively.

**Fig 2:**
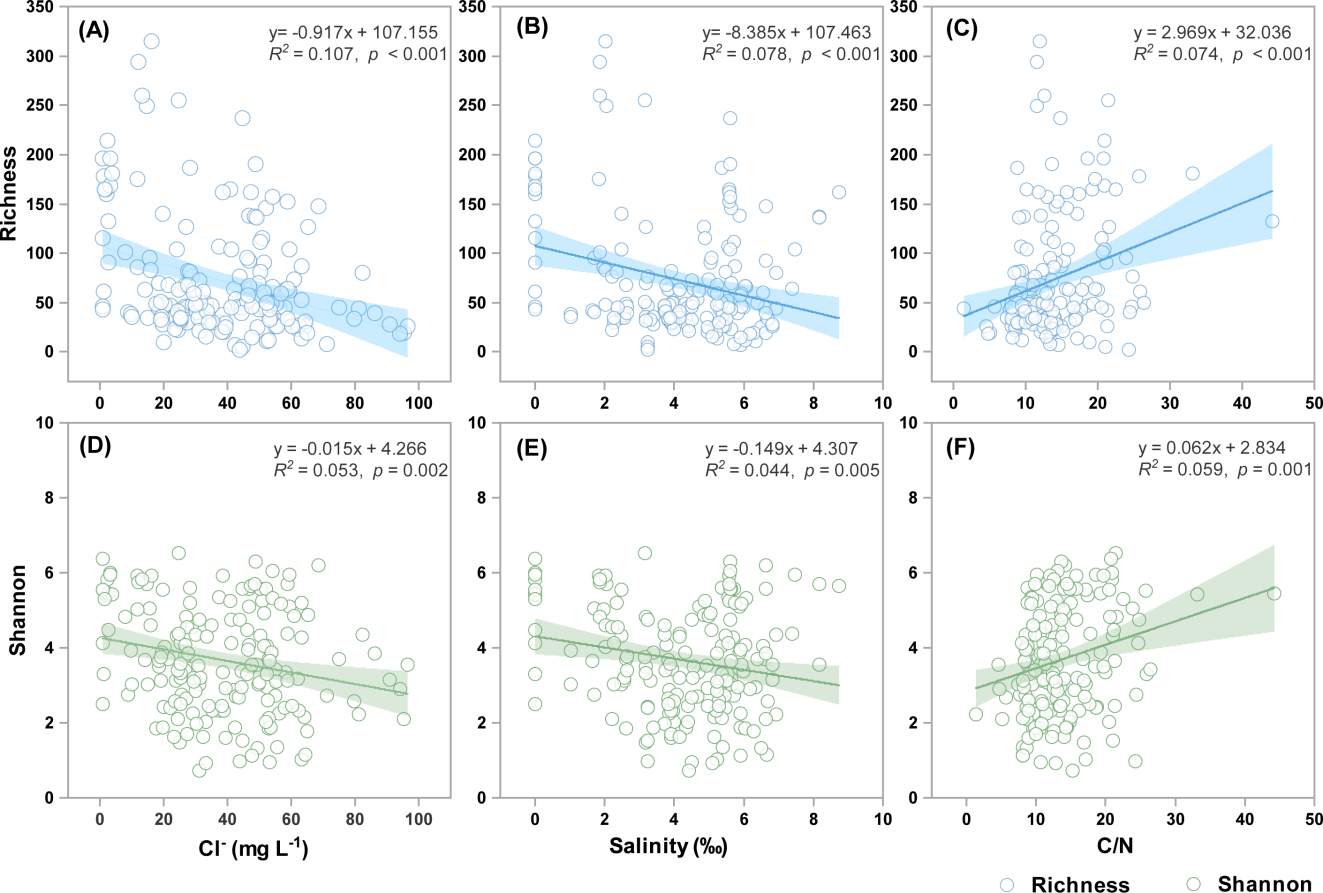
Correlation analysis between the richness (**A–C**) or Shannon diversity (**D–F**) and soil Cl^−^, salinity, or C/N ratio across all sampling sites. Solid lines denote the ordinary least-squares linear regressions. The shaded area indicates the 95% confidence interval of the regression models.

After quality control and the removal of non-protist eukaryotic sequences, a total of 8,150,898 high-quality reads were obtained from 179 sediment samples (3 samples were excluded due to PCR failure and 7 due to extremely low protistan sequence counts). These reads were clustered into 3,391 protistan ASVs. Neither the invasion of *S. alterniflora* nor its subsequent conversion into aquaculture ponds significantly affected protistan richness or Shannon diversity (Fig. 1A and C). Random forest analysis identified salinity, Cl^−^, and C/N ratio as the primary drivers of protistan alpha diversity (*P* < 0.05) (Fig. 1B and D), with richness and Shannon diversity showing negative correlations with Cl^−^ (*P* < 0.01) and salinity (*P* < 0.01), and positive correlations with the C/N ratio (*P* < 0.01) (Fig. 2). In addition, SOC (*P* < 0.05) and sand content (*P* < 0.01) also emerged as important predictors of protistan diversity (Fig. 1B and D).

**Fig 3:**
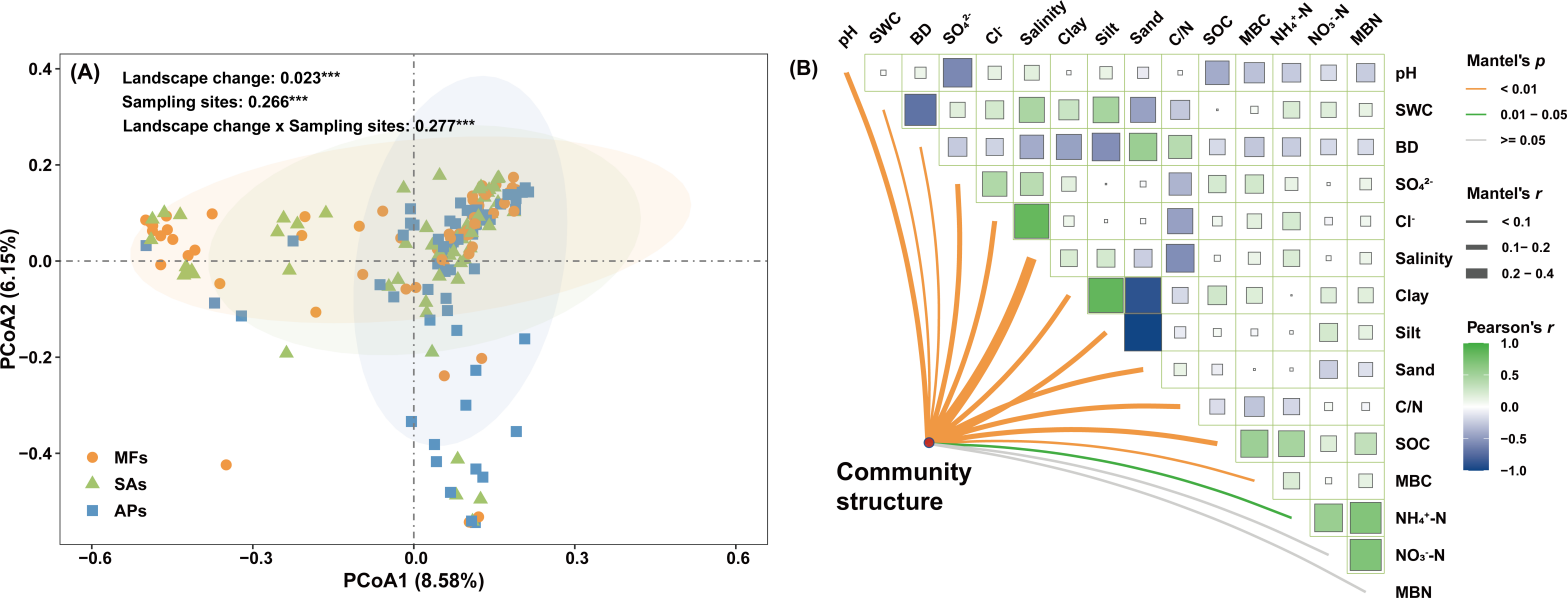
Principal coordinate analysis (PCoA) of protistan communities based on the Bray-Curtis distances across the three wetland habitat types (**A**). Mantel test showing the relationships between the protistan community structure and environmental factors across the three wetland habitat types (**B**). The numbers in PCoA plot indicate the *R*^2^ values, while asterisks *** represent statistically significant at 0.001 probability level as revealed by PERMANOVA. Edge width of plot B is proportional to Mantel’s *r* value, and the edge colors indicate statistical significance. Pairwise correlation coefficients of environmental factors are shown with color gradients. BD, bulk density; MBC, microbial biomass carbon; MBN, microbial biomass nitrogen; SOC, soil organic carbon; SWC, sediment water content.

PCoA showed partial overlap of protistan community structures across the three habitat types (Fig. 3A). However, PERMANOVA results indicated that 56.6% of the total variation in community composition could be explained by sampling site, habitat type, and their interaction (*P* = 0.01) (Fig. 3A). Despite this, PERMANOVA, ANOSIM, and MRPP analyses consistently showed significant differences in protistan community composition between aquaculture ponds and both mudflats and *S. alterniflora* marshes, whereas differences between mudflats and *S. alterniflora* marshes were relatively minor (Table 1). Mantel test results suggested that sediment salinity was the most influential factor shaping the protistan community structure (Mantel *r* = 0.227, *P* < 0.001) (Fig. 3B). In addition to sediment salinity, Cl^−^ concentration, soil texture (sand, silt, and clay fractions), pH, C/N, SOC, and SO_4_^2−^ concentration emerged as the significant predictors of the protistan community structure variation (*P* < 0.001). At the taxonomic level, *Tubulinea* dominated protistan assemblages in mudflats (relative abundance 30.78% ± 4.19%) and *S. alterniflora* marshes (relative abundance 32.34% ± 4.41%), while *Cercozoa* was most abundant in aquaculture ponds (relative abundance 28.41% ± 2.66%) (Fig. S2). The invasion of *S. alterniflora* did not significantly change the relative abundance of major protistan taxa, while aquaculture pond reclamation substantially reshaped the community, with *Cercozoa* increasing significantly (*P* < 0.01) from 15.64% to 28.41%. In addition, the relative abundances of *Chrompodellids*, *Haptophyta,* and *Kathablepharida* increased significantly (*P* < 0.05), whereas *Breviatea* and *Tubulinea* decreased significantly (*P* < 0.05) (Fig. S2).

**TABLE 1:** PERMANOVA, ANOSIM, and MRPP analyses of the community structure of protists among three wetland habitat types^*a*^

Pairwise comparison	PERMANOVA		ANOSIM		MRPP	
	*R* ^2^	*P*	*R*	*P*	*A*	*P*
MFs vs. SAs	0.012	0.071	0.016	0.083	0.002	0.042
MFs vs. APs	0.021	<0.001	0.063	<0.001	0.008	<0.001
SAs vs. APs	0.020	<0.001	0.058	0.002	0.007	<0.001

^*a*^ APs, aquaculture ponds; MFs, mudflats; SA, *S. alterniflora* marshes. The *A*-value represents the degree of consistency within the group.

### Functional groups of protists and their environmental drivers

**Fig 4:**
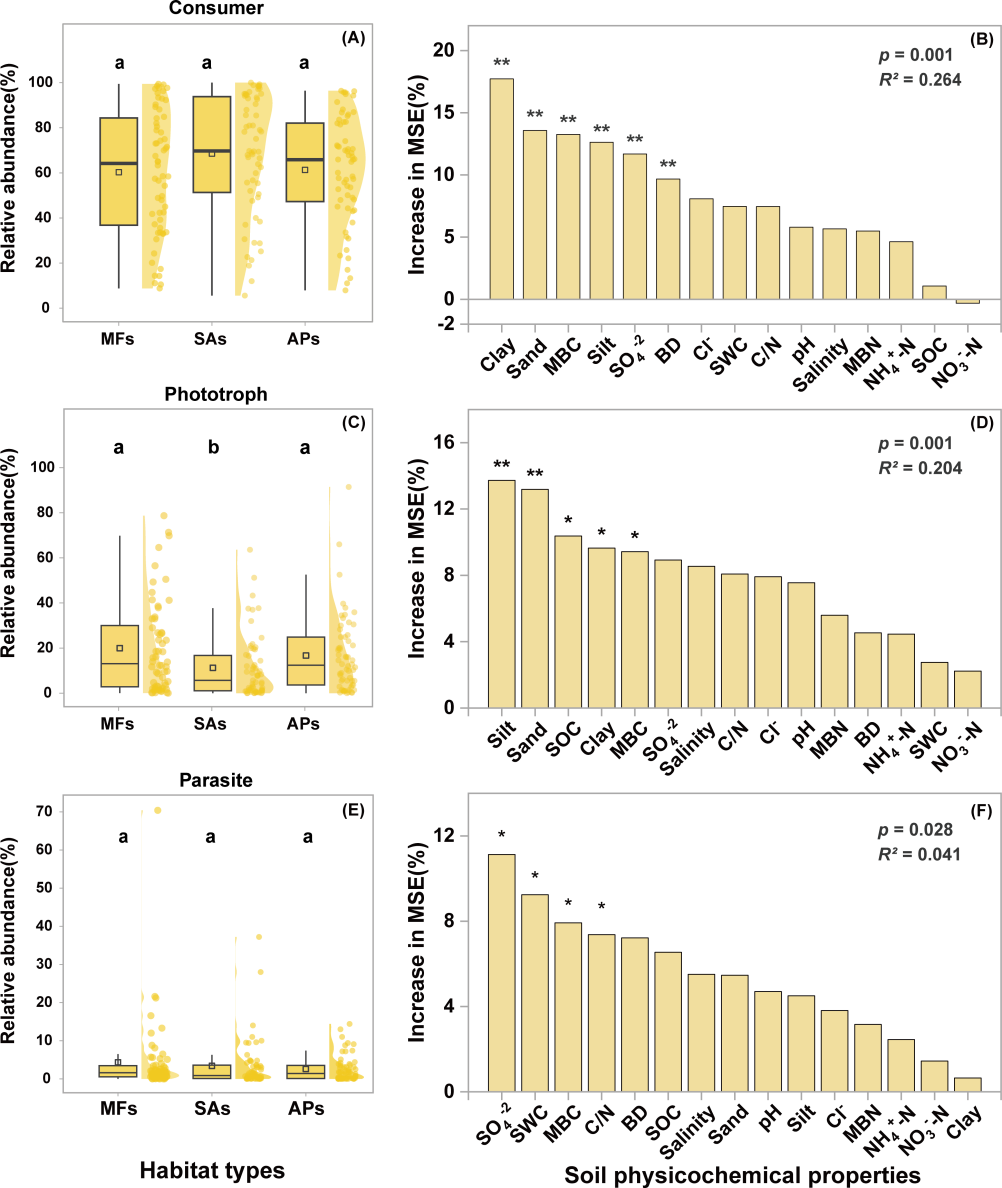
Box-normal plots showing the distribution of the consumers (**A**), phototrophs (**C**), and parasites (**E**) in the three wetland habitat types. Random forest analysis showing the importance of environmental factors in influencing the consumers (**B**), phototrophs (**D**), and parasites (**F**). Different lowercase letters above the boxes indicate significant differences between wetland habitat types (*P* < 0.05). Asterisks indicate levels of significance (*P < 0.05; **P < 0.01). BD, bulk density; MBC, microbial biomass carbon; MBN, microbial biomass nitrogen; SOC, soil organic carbon; SWC, sediment water content. APs, aquaculture ponds; MFs, mudflats; SA, *S. alterniflora* marshes.

Protists were functionally categorized into consumers, phototrophs, and parasites. Consumers, which include predatory protists, were the dominant functional group across all three habitat types (Fig. 4), comprising 60.30%, 68.54%, and 61.31% of the communities in mudflats, *S. alterniflora* marshes, and aquaculture ponds, respectively (Fig. 4A). The relative abundances of consumers and parasites did not differ significantly among the three habitats (Fig. 4A and E). However, the relative abundance of phototrophs significantly declined from 19.96% to 11.20% following *S. alterniflora* invasion (*P* < 0.01) and then increased to 16.70% after aquaculture reclamation (*P* < 0.05) (Fig. 4C).

**Fig 5:**
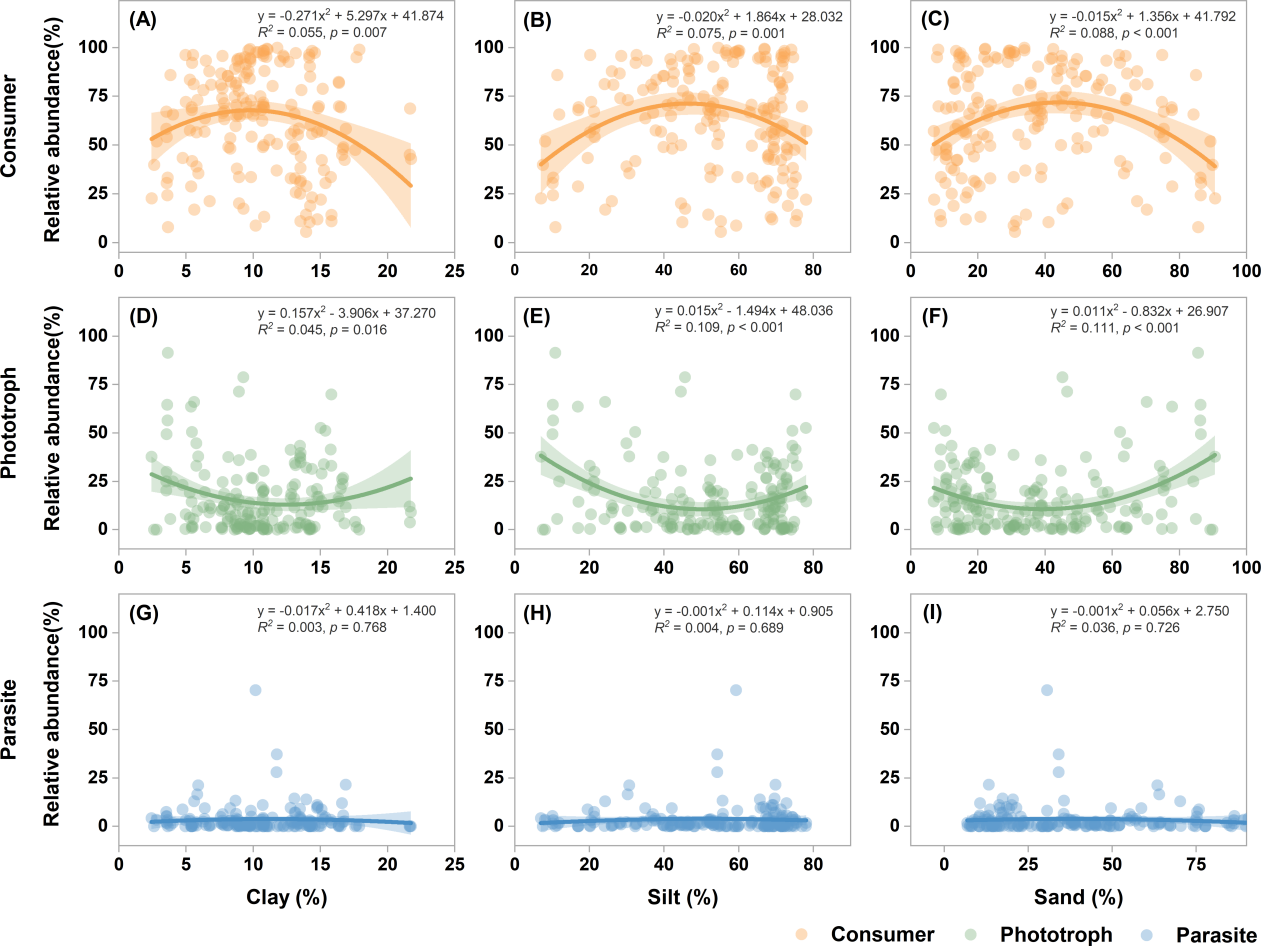
The relationship between the relative abundance of consumers (**A–C**), phototrophs (**D–F**), and parasites (**G–I**) and the proportions of clay, silt, and sand particles across all sampling sites. Solid lines denote the ordinary least-squares linear regressions. The shaded area indicates the 95% confidence interval of the regression models.

Random forest analysis identified sediment grain size, particularly the proportions of clay, sand, and silt, as the strongest predictors of the relative abundances of consumers and phototrophs, while SO_4_^2−^ and SWC were the key predictors for parasites (Fig. 4). Quadratic regression revealed significant nonlinear relationships between grain size and the relative abundances of functional groups (Fig. 5). Specifically, the relative abundance of consumers exhibited a hump-shaped relationship, increasing and then decreasing with higher proportions of clay, silt, or sand. Conversely, phototroph abundance showed a U-shaped trend, initially decreasing and then increasing. Notably, the proportions of silt and sand particles exerted a stronger influence on functional group composition than did clay content.

### Distance-decay relationships and community assembly mechanisms

**Fig 6:**
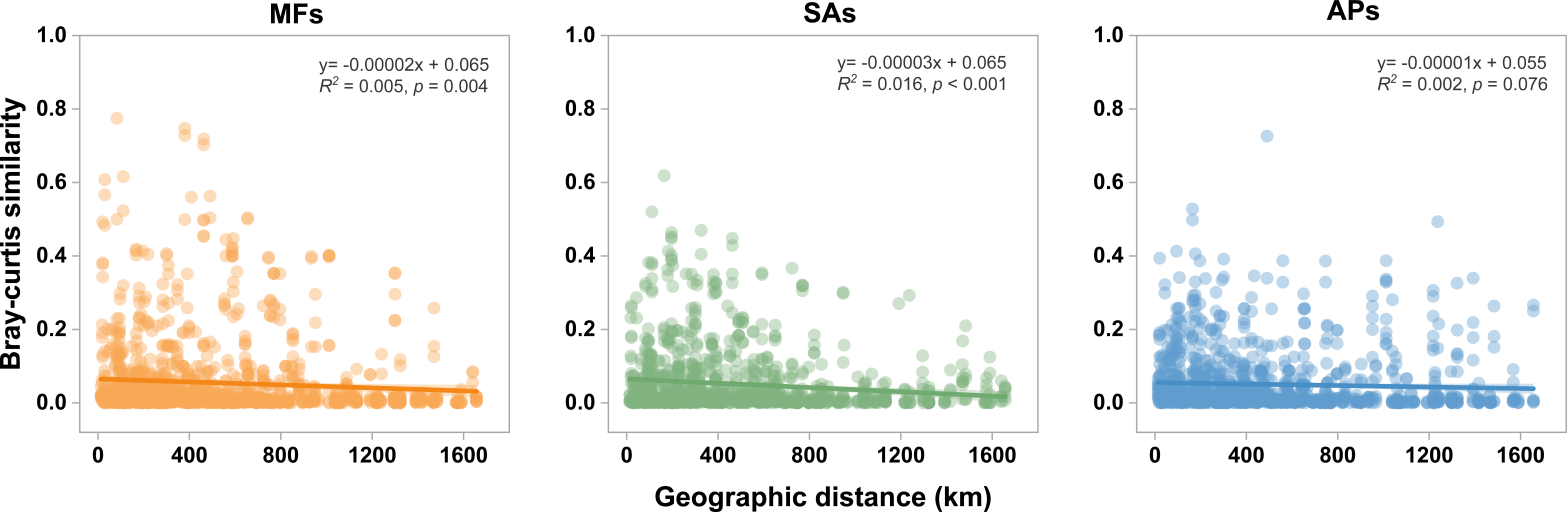
Distance-decay curves showing the relationships between the Bray-Curtis similarity of protistan communities and geographic distances in the three wetland habitat types. Solid lines denote the ordinary least-squares linear regressions. The shaded area indicates the 95% confidence interval of the regression models.

Distance-decay relationships (DDR) analysis revealed significant spatial turnover in protistan communities in mudflats and *S. alterniflora* marshes (*P* < 0.05), but not in aquaculture ponds (*P* = 0.076) (Fig. 6). The slope of the DDR regression for *S. alterniflora* marshes (slope: 0.00003) was steeper than that for mudflats (slope: 0.00002), suggesting enhanced spatial turnover in protistan communities following plant invasion.

To assess the underlying mechanisms of community assembly, we calculated the modified stochasticity ratio (MST) (52). MST values for all three habitats were below 50%, indicating that deterministic processes predominantly governed protistan community assembly (Fig. S3). Moreover, a Mann-Whitney *U* test showed that MST values were significantly lower in *S. alterniflora* marshes than in both mudflats (*P* < 0.001) and aquaculture ponds (*P* < 0.05), suggesting a stronger influence of deterministic factors in *S. alterniflora* marsh habitats compared to the other two.

## DISCUSSION

### Effect of habitat change on the diversity and community structure of protists

*S. alterniflora* invasion and aquaculture reclamation represent two widespread and impactful forms of habitat modification in coastal wetlands, with well-documented effects on soil properties and ecosystem functioning (53–55). In this study, we found that these habitat changes significantly altered several key soil parameters, including SOC, NH_4_^+^-N, NO_3_^−^-N, MBN, and SO_4_^2−^ concentrations. However, contrary to our initial hypothesis, these changes did not significantly influence protistan richness or Shannon diversity. Instead, both random forest modeling and linear correlation analyses identified Cl^−^, salinity, and C/N ratio as the primary factors associated with protistan diversity, rather than the aforementioned nutrient parameters. Specifically, richness and Shannon diversity were negatively correlated with Cl^−^ and salinity, and positively correlated with C/N ratio. Among these factors, salinity emerged as the most influential predictor, consistent with earlier studies in coastal wetland ecosystems (56, 57). Salinity and Cl^−^ fluctuations can directly affect protists by altering osmotic pressure and intracellular ion balance, thereby influencing cell metabolism, motility, and survival (58, 59). In addition, salinity may indirectly reshape protistan diversity by modifying bacterial and fungal community composition, altering prey availability, and restructuring microbial food-web interactions (60, 61). These indirect effects can influence predator-prey dynamics, trophic niche breadth, and consequently, community diversity. The soil C/N ratio also showed a clear positive relationship with protistan diversity. Higher C/N ratios generally favor fungal over bacterial dominance (62), leading to shifts in the quality and size distribution of potential prey for protists. Such prey community restructuring can alter protistan feeding strategies, from bacterivory-dominated diets to more mixed or fungivory-influenced diets, thereby promoting niche differentiation and sustaining higher diversity levels (63). The absence of significant diversity shifts across habitats in our study is likely due to the non-significant salinity differences between habitat types (Table S1).

Unlike diversity metrics, protistan community structure was markedly more responsive to environmental variation, a pattern also observed in previous microbial ecology studies (64, 65). While habitat change significantly influenced the overall community composition, the effects of sampling site and its interaction with habitat type were stronger than habitat type alone (Fig. 3A). This highlights the importance of localized environmental heterogeneity, even within the same habitat classification, in shaping protistan assemblages. Notably, protistan communities in aquaculture ponds differed significantly from those in mudflats and *S. alterniflora* marshes (Table 1). Among the environmental factors measured, salinity emerged as the primary determinant of protistan community structure based on Mantel test results, consistent with findings from mangrove and other anaerobic wetland systems (66). Salinity fluctuations may selectively favor certain protistan taxa while suppressing others, thereby reshaping community structure. This is supported by Bodur et al. (46), who reported concurrent shifts in both alpha and beta diversity of soil protists along salinity gradients. Mechanistically, salinity may influence protists directly by affecting osmotic pressure and intracellular ion balance (67), and indirectly by modifying microbial prey availability and interactions (60, 61). Therefore, salinity not only serves as a key environmental filter but also acts as a sensitive ecological indicator for predicting protistan diversity and community responses to habitat transformation in coastal wetlands (46). Taxonomic shifts were also evident at the group level. *Tubulinea*, which was the dominant lineage in mudflats (relative abundance 30.78%) and *S. alterniflora* marshes (relative abundance 32.34%), showed a marked decline in relative abundance following aquaculture reclamation (relative abundance 14.99%). Conversely, *Cercozoa* significantly increased and became the most dominant group in aquaculture pond environments (relative abundance 28.41%) (Fig. S1 and S2). This shift mirrors observations in other aquaculture systems (27, 64) and likely reflects the ecological versatility of *Cercozoa*. Their broad ecological amplitude, nutritional flexibility, and high tolerance to fluctuating physicochemical conditions, including elevated SO_4_^2−^ levels, enable them to thrive in the altered environments created by aquaculture activities (3).

### Effect of habitat change on protistan functional groups

Protists play diverse functional roles in soil ecosystems, with consumers generally dominating terrestrial communities due to their pivotal role in regulating microbial turnover and nutrient cycling (3, 8). Consistent with these findings, our study showed that consumers represented the most abundant functional group across all three habitat types. Interestingly, we observed that *S. alterniflora* invasion led to a significant reduction in the relative abundance of phototrophs, whereas other functional groups, including consumers and parasites, showed no statistically significant changes following habitat modification. A plausible explanation is that the dense aboveground biomass of *S. alterniflora* reduces light penetration into the soil, thus limiting the capacity for phototrophic activity in the surface sediment (68, 69).

Among the measured environmental variables, sediment particle size emerged as the most influential factor regulating the relative abundances of consumers and phototrophs. This finding aligns with prior studies demonstrating that soil texture strongly affects microbial spatial distribution and activity through its influence on pore structure, water retention, and oxygen diffusion (65, 70, 71). Specifically, consumer-microbe interactions largely occur within soil pores (72, 73), and the physical characteristics of these pore spaces can either facilitate or constrain predatory behaviors. Sediments rich in clay and silt typically contain smaller pores that harbor high microbial biomass, offering abundant prey for consumers (74). However, the restricted pore space can limit protistan motility and access to prey organisms (75, 76). Conversely, sandy sediments possess larger pores that enhance mobility but often lack sufficient microbial prey density. Beyond these direct physical constraints, soil texture can also indirectly influence protistan consumers by shaping the abundance and diversity of their prey. Bacteria and fungi exhibit distinct particle-size preferences and attachment patterns, fungi often associate with larger pores and particulate organic matter, whereas many bacteria preferentially colonize fine particles such as clay and silt (77, 78). Such spatial partitioning can stabilize microbial community structure and generate heterogeneity in prey availability across texture gradients, which, in turn, drives compositional differences in protistan consumer communities (45). Therefore, both the physical accessibility of pore networks and the texture-mediated structuring of bacterial and fungal communities likely contribute to the observed quadratic relationship between consumer abundance and sediment texture composition. Future research integrating detailed bacterial and fungal community profiling with protistan functional traits would provide deeper insights into the mechanisms underpinning these texture-consumer linkages.

For phototrophs, sediment structure also plays a crucial role but through different mechanisms. Clay and silt particles provide a large surface area that serves as a substrate for attachment and may offer protection from grazers (79). In contrast, sandy sediments, due to their higher porosity, allow for deeper light penetration, which supports photosynthetic activity in subsurface environments (74). Therefore, both the physical and optical properties of the sediment matrix collectively shape the distribution of phototrophic protists. In contrast, the relative abundance of parasitic protists showed no significant association with particle size. This may be attributed to their dependence on host availability rather than on abiotic sediment properties. As parasites rely on specific host organisms for survival, their population dynamics are likely constrained by the presence and density of susceptible hosts rather than by environmental factors such as texture or nutrient availability (80).

### Effect of habitat change on DDRs and assembly processes of protistan communities

Our study revealed that *S. alterniflora* invasion significantly steepened the distance-decay curves of protistan communities compared to mudflats (Fig. 6), a pattern consistent with previous observations in bacterial communities (24). The DDR framework quantifies how community similarity declines with increasing geographical distance, providing insights into spatial turnover patterns and the underlying ecological processes. Although the explanatory power (*R*^2^) of the DDR models was relatively low, this is expected because DDRs are calculated from all pairwise comparisons among numerous sampling sites, inherently introducing high variability into the relationship. Nonetheless, the observed differences in DDR slopes remain ecologically meaningful, reflecting consistent changes in spatial turnover rates among habitats rather than attempting to predict community similarity from distance with high precision (81). In open mudflat habitats, strong tidal influences likely promote environmental homogenization across spatial scales, thereby weakening spatial turnover in microbial communities (41, 82). In contrast, *S. alterniflora* marshes increase spatial heterogeneity through complex belowground and aboveground inputs, including root exudates and litter, which contribute to the formation of more variable microhabitats (69, 83). Such heterogeneity enhances spatial turnover, steepening DDR slopes.

To further disentangle the processes governing community assembly, we applied the modified stochasticity ratio (MST), which quantitatively partitions assembly processes into deterministic versus stochastic components. This approach complements DDR analysis by revealing whether observed spatial patterns are primarily driven by niche-based environmental selection or by random dispersal and drift (52). MST analysis indicated that deterministic processes played a more prominent role in shaping protistan community assembly in *S. alterniflora* marshes than in mudflats (Fig. S3). The introduction of this invasive plant likely disrupted native habitats and restructured local microenvironments, increasing niche differentiation and thereby amplifying environmental selection (57, 69). This shift underscores the role of plant-mediated environmental heterogeneity in enhancing deterministic community assembly processes.

In contrast, aquaculture ponds did not exhibit a significant DDR (Fig. 6), suggesting a breakdown of spatial turnover in protistan communities under anthropogenic modification. This pattern mirrors findings in agricultural soils where frequent tillage and human management reduced spatial differentiation in bacterial communities (84). The conversion of coastal wetlands into aquaculture ponds creates semi-enclosed systems characterized by restricted natural dispersal and intensive anthropogenic inputs. Such management practices, often standardized across sites, increase environmental homogeneity, thereby weakening the biogeographical structuring of microbial communities (48). Interestingly, although the absence of a significant DDR in aquaculture ponds might suggest a predominance of stochastic processes, our MST analysis indicated that deterministic processes still dominated community assembly. This apparent contradiction can be reconciled by considering the role of strong environmental filtering. Specifically, SO_4_^2−^ concentrations were markedly elevated in aquaculture pond sediments, likely due to inputs associated with high-density aquaculture and associated anaerobic conditions. SO_4_^2−^ has been shown to exert selective pressure on microbial communities (85, 86). Indeed, Wu et al. (87) found that SO_4_^2−^ concentration was a key driver of deterministic assembly in bacterial communities across large-scale aquaculture systems. Our findings extend this understanding to protistan communities, suggesting that elevated SO_4_^2−^ acts as a strong environmental filter, overriding the homogenizing effects of human management and reinforcing deterministic community assembly processes. Collectively, these results demonstrate that habitat changes not only alter the spatial structure of protistan communities but also reshape the underlying ecological processes driving community assembly.

Our study provides comprehensive insights into the impacts of *S. alterniflora* invasion and subsequent reclamation into aquaculture ponds on sediment protistan communities along China’s southeastern coast. Although these habitat transitions did not significantly alter protistan alpha diversity, they substantially reshaped community composition and functional group structure. Specifically, phototrophs declined markedly following *S. alterniflora* invasion, while cercozoan taxa became more dominant in aquaculture ponds. Random forest and Mantel analyses identified sediment salinity, Cl^−^, C/N ratio, SO_4_^2−^, and grain size distribution as key environmental factors influencing both diversity and community structure. Moreover, spatial turnover of protistan communities increased following plant invasion, as evidenced by steeper distance-decay slopes in *S. alterniflora* marshes. Community assembly was predominantly shaped by deterministic processes across all habitats, with *S. alterniflora* marshes showing the strongest deterministic signal. These findings suggest that coastal habitat transformation imposes strong environmental filtering on protistan communities, thereby altering their ecological roles and functional potentials. Taken together, this study highlights the ecological sensitivity of protists to anthropogenic habitat modification and underscores their potential as bioindicators for coastal wetland health and resilience.

## MATERIALS AND METHODS

### Study sites and sample collection

**Fig 7:**
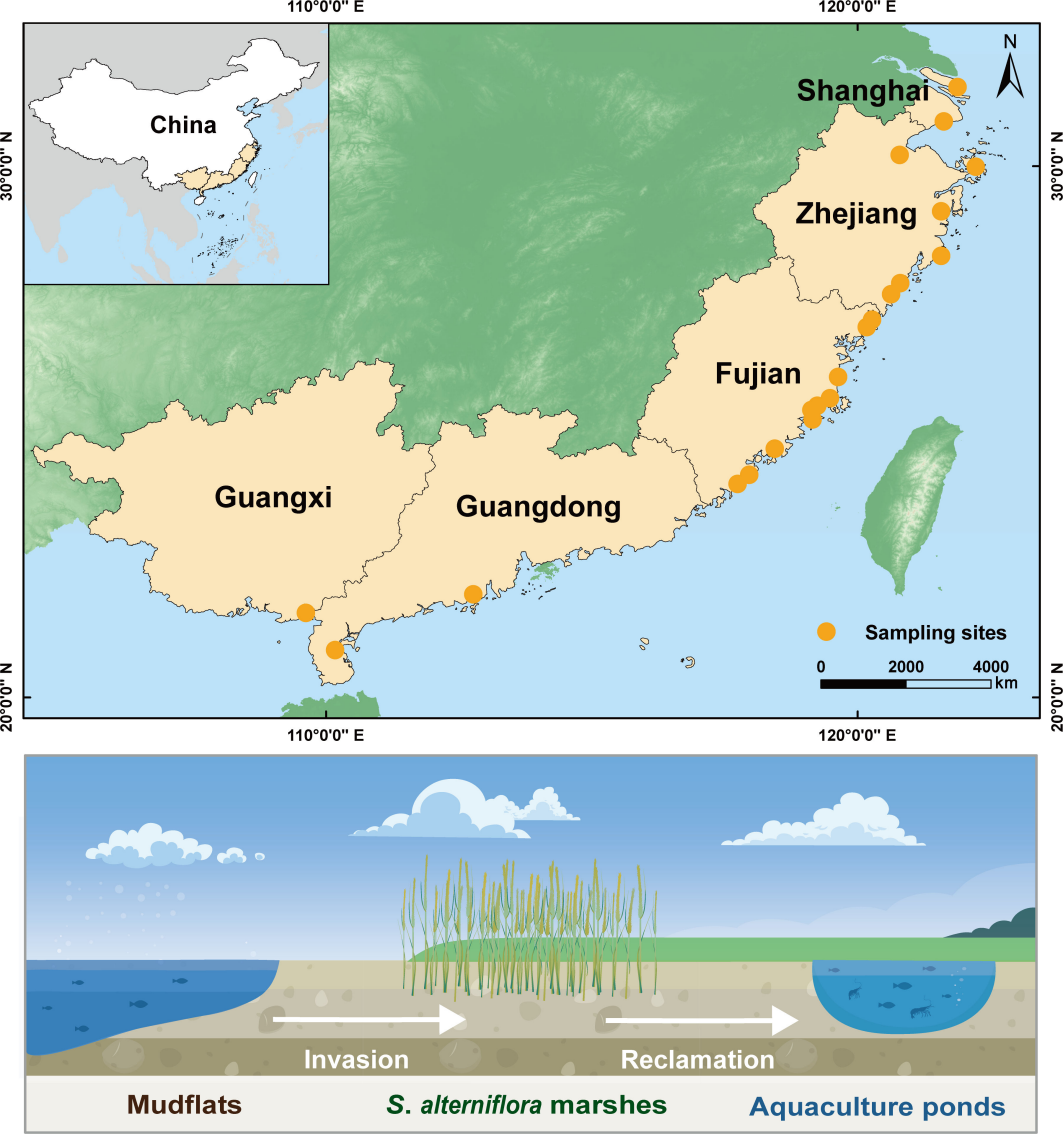
Locations of the study area and 21 sampling sites across the coastal regions in southeastern China. The map was created using ArcGIS 10.8.

Sediment samples were collected from 21 coastal wetland sites spanning tropical and subtropical climatic zones in China (20°42′ N to 31°51′ N; 109°11′ E to 122°11′ E) (Fig. 7). These sites were located across five southern coastal provinces: two in Shanghai (SH), six in Zhejiang (ZJ), nine in Fujian (FJ), three in Guangdong (GD), and one in Guangxi (GX). The region is characterized by a tropical to subtropical monsoon climate, with mean annual temperatures ranging from 11.0 to 23.0°C and annual precipitation between 100 and 2,200 mm. As of 2014, the total area of coastal wetlands in these five provinces reached approximately 2.58 × 10^6^ hectares, accounting for 44.5% of China’s coastal wetlands (53). Tidal flats covered around 3.6 × 10^5^ hectares, representing 42.4% of the national total (88). In recent decades, large portions of these tidal flats have undergone substantial transformation, initially colonized by the invasive plant *S. alterniflora*, and subsequently reclaimed for aquaculture use. Within the surveyed region, *S. alterniflora* marshes and aquaculture ponds occupy 3.34 × 10^4^ and 5.31 × 10^5^ hectares, respectively, accounting for 61.2% of China’s *S. alterniflora* marsh area (89) and 36.9% of its aquaculture pond area (90).

Field surveys and sediment sampling were conducted between December 2019 and January 2020. Three distinct habitat types were identified at each sampling site, mudflats (MFs), *S. alterniflora* marshes (SAs), and aquaculture ponds (APs) based on a combination of remote sensing data and ground-truth surveys. At each site, three replicate plots were sampled per habitat type, yielding a total of 189 sediment samples (21 sampling sites × 3 habitats × 3 plots). Replicate sampling plots within each habitat were located within a 500 m radius to minimize local environmental variation. Each sampling plot covered an area of 10 m × 10 m, with a minimum spacing of 50 m between adjacent plots to minimize cross-contamination and ensure spatial independence. Surface sediments (0–20 cm depth) were collected using a stainless-steel corer (5 cm inner diameter, 1.5 m in length). Samples were immediately transferred into sterile plastic bags, stored on ice during transport, and delivered to the laboratory for further processing. Upon arrival, each sample was homogenized and divided into two portions: one stored at 4°C for physicochemical analysis, and the other preserved at −80°C for molecular analyses.

### Physicochemical analysis

Sediment properties were analyzed following the methods described in Lin et al. (35). After removing visible plant residues and stones, fresh sediment samples were sieved through a 2 mm mesh. Sediment pH was measured in a 1:2.5 (wt/vol) sediment-to-deionized water slurry using an Orion 868 pH meter (Thermo Fisher Scientific, USA). Salinity was measured at 1:5 (wt/vol) ratio by a Eutech Instruments-Salt6 salinity meter (Thermo Fisher Scientific, USA). Sediment water content (SWC) and bulk density (BD) were determined by oven-drying fresh samples at 105°C for 48 h (91, 92). Grain size distribution was measured using laser diffraction (Malvern Scientific Instruments, Suffolk, UK). Porewater inorganic nitrogen (NH_4_^+^-N and NO_3_^−^-N) was extracted by 2 M KCl (93, 94) and quantified with a continuous flow analyzer (Skalar Analytical SAN^++^, Netherlands). Sediment samples were freeze-dried, homogenized, and ground to a fine powder. Subsequently, approximately 1 g of this prepared sample was analyzed for total carbon (TC) and total nitrogen (TN) using an elemental combustion analyzer (Elementar Vario MAX CN, Germany). The C:N ratio was calculated as the ratio of TC to TN. To measure soil organic carbon (SOC), approximately 3 g of freeze-dried sample was screened, weighed, and extracted in 1 M hydrochloric acid (HCl) solution for 24 h to remove the inorganic carbon, then oven-dried at 60°C (95), and subsequently analyzed with the combustion analyzer (Elementar Vario MAX CN, Germany). Microbial biomass carbon (MBC) and microbial biomass nitrogen (MBN) were quantified using the chloroform fumigation-extraction method (96). Concentrations of sulfate (SO_4_^2−^) and chloride (Cl^−^) in sediment porewater were measured by ion chromatography (Dionex 2100, USA) (97).

### DNA extraction, amplification, and sequencing

Genomic DNA was extracted from 0.5 g of each sediment sample using the FastDNA SPIN Kit for Soil (MP Biomedicals, USA), following the manufacturer’s protocol. DNA quality and concentration were assessed via agarose gel electrophoresis and spectrophotometric analysis using a spectrophotometer (NanoDrop Technologies, Wilmington, USA). The protistan 18S rRNA gene was amplified using the universal eukaryotic primer pair F-TAReuk454FWD1/R-TAReukREV3 (98). PCR products were purified, pooled in equimolar concentrations, and subjected to paired-end sequencing on the Illumina MiSeq platform by Majorbio (Shanghai, China).

Bioinformatics analysis was mainly performed using QIIME 2 (99). Primers were removed using cutadapt (v 1.9) (100). Barcodes and adaptors were removed from demultiplexed sequences using fastp (101). Reads with quality scores below 20 and lengths under 50 bp were filtered out. Paired-end reads were merged using FLASH (102). Sequences were then denoised, and chimeras were removed using the DADA2 plugin (103). Sequences with 100% similarity were clustered into amplicon sequencing variants (ASVs). The taxonomic classification of ASVs was conducted using the Protist Ribosomal Reference Database (PR2, v 5.0.1) (104). The nonprotist sequences, including Rhodophyta, Streptophyta, Metazoa, Fungi, Opisthokonta_X, and unclassified eukaryotes, were removed (47). Based on earlier studies, we classified the protists into consumers, phototrophs, parasites, and unassigned based on protistan functional traits (3, 6).

### Statistical analysis

To explore the relationships between protistan communities and edaphic factors across the three habitat types, a series of statistical analyses were performed with clearly defined purposes, software, and functions. Alpha diversity indices, including species richness and Shannon diversity, were calculated using the *diversity* and *estimateR* functions in the “vegan” package in R (v 4.3.4). Differences in sediment physicochemical properties, protistan alpha diversity indices, the relative abundance of protists at the subdivision level, and the relative abundance of functional groups among habitats were evaluated via one-way analysis of variance (ANOVA) using SPSS 26.0 (IBM Corp., Armonk, NY, USA). Data normality and variance homogeneity were tested using the Shapiro-Wilk test and Levene’s test, respectively. *Post hoc* comparisons for ANOVA were performed using the least significant difference (LSD) test. Non-conforming data were log-transformed to meet model assumptions. When assumptions could not be met, the non-parametric Kruskal-Wallis test was applied, followed by pairwise Wilcoxon rank-sum tests with Benjamini-Hochberg (BH) correction for multiple comparisons (105).

To assess the relationships between protistan alpha diversity, functional group composition, and sediment properties, random forest analysis was conducted using the “randomForest" package (106), with variable importance evaluated via permutation-based significance testing. Ordinary least squares (OLS) regression models were fitted using the *lm* function in R to explore linear relationships between key physicochemical variables and protistan diversity.

For community composition analyses, the ASV matrix was Hellinger-transformed using the *decostand* function in the “vegan” package. Principal coordinate analysis (PCoA) was performed using the *pcoa* function in the “ape” package. Community dissimilarity was calculated using Bray-Curtis distances, and the effects of habitat type and sampling site were tested using permutational multivariate analysis of variance (PERMANOVA; *adonis* function), analysis of similarities (ANOSIM; *anosim* function), and multi-response permutation procedure (MRPP; *mrpp* function), all with 999 permutations in the “vegan” package. Mantel tests were performed to assess correlations between environmental variables and community dissimilarity with the *mantel* function in the “vegan” package.

Distance-decay relationships (DDRs) were quantified by regressing Bray-Curtis similarity (1—Bray-Curtis dissimilarity) against log-transformed geographic distance (calculated via the Haversine formula) using OLS regression in R, with permutation tests (999 permutations) to assess slope significance. To infer the relative importance of deterministic vs stochastic processes in shaping protistan community assembly, the modified stochasticity ratio (MST) was calculated using the “NST” package (52). The MST is calculated under the assumption that deterministic processes drive the community to be either more similar or dissimilar than the null expectation (107). MST is a null model-based index of ecological stochasticity computed here under the “PF” null model with 1,000 randomizations, using both the Jaccard and Bray-Curtis indices. MST values > 0.5 indicate stochastic processes dominate, whereas values < 0.5 suggest a greater influence of deterministic factors. Additionally, nonparametric Mann-Whitney *U* tests were used to compare the significant difference of MST values among habitat types.

## Data Availability

Raw sequence data were submitted to the NCBI Sequence Read Archive under BioProject PRJNA1265662.

## SUPPLEMENTAL MATERIAL

Supplemental material aem.01661-25-s0001.docx Table S1; Figures S1 to S3.

ASM does not own the copyrights to Supplemental Material that may be linked to, or accessed through, an article. The authors have granted ASM a non-exclusive, world-wide license to publish the Supplemental Material files. Please contact the corresponding author directly for reuse.
