# Supplementary material for: Habitat transformation reshapes protistan community composition and assembly processes in coastal wetlands of southeastern China
Source: Appl Environ Microbiol. 2025 Oct 9;91(11):e01661-25. doi: 10.1128/aem.01661-25 (PMC12628693; doi:10.1128/aem.01661-25)

**Supplementary Materials**

**Table S1** Physicochemical properties of the sediments and porewater in the three wetland habitat types.

|  | MFs | SAs | APs |
| --- | --- | --- | --- |
| pH | 7.99 ±0.06a | 7.99 ±0.06a | 7.82 ±0.11a |
| SWC (%) | 43.1 ±1.33a | 47.1 ±1.38ab | 47.8 ±1.70b |
| BD (g cm^-3^) | 1.29 ±0.02b | 1.26 ±0.02ab | 1.23 ±0.02a |
| SO_4_^2-^(mg L^-1^) | 8.90 ±0.63a | 9.13 ±0.50a | 17.5 ±1.40b |
| Cl^-^ (mg L^-1^) | 36.8 ±2.15a | 40.9 ±2.23a | 37.8 ±3.43a |
| Salinity (‰) | 3.96 ± 0.20a | 4.54±0.23a | 4.21 ±0.31 a |
| Clay (%) | 10.4 ±0.47a | 10.9 ±0.49a | 10.5 ±0.57a |
| Silt (%) | 54.1 ±2.29a | 52.7 ±2.41a | 50.1 ±2.56a |
| Sand (%) | 35.5 ±2.69a | 36.4 ±2.86a | 39.4 ±3.06a |
| C/N | 14.0 ±0.60a | 13.9 ±0.62a | 12.9 ±0.83a |
| SOC (g kg^-1^) | 6.81 ±0.22a | 10.0 ±0.54c | 8.48 ±0.49b |
| MBC (mg kg^-1^) | 326 ±23.7a | 343 ±28.6a | 385 ±33.5a |
| NH_4_^+^-N (mg kg^-1^) | 13.3 ±0.82a | 25.0 ±1.40c | 16.9 ±1.03b |
| NO_3_^-^-N (mg kg^-1^) | 1.25 ±0.03a | 1.85±0.11b | 1.45 ±0.08a |
| MBN (mg kg^-1^) | 12.7 ±0.65a | 26.6 ±1.99c | 16.8 ±0.82b |

SWC: soil water content; BD: bulk density; SOC: soil organic carbon; MBC: microbial biomass carbon; MBN: microbial biomass nitrogen. APs, aquaculture ponds; MFs, mudflats; SA, *S. alterniflora* marshes. Different lowercase letters within the same row indicate significant differences at *p* < 0.05. Data are after Yang et al. (2023) for reference and review only.

**Figure captions**

**Fig. S1** The soil protistan community composition at the subdivision level between three wetland habitat types.

**Fig. S2** Changes in the relative abundance of protists at the subdivision level under habitat variation. The difference in relative abundance between protists at the subdivision level under habitat change was plotted as response ratios (log2 fold change). Negative values (depicted by blue lines) indicate a decrease in the abundance of species after habitat changes. Positive values (depicted by orange lines) indicate an increase in the abundance of species after habitat changes. Error bars indicate standard errors (n = 21). Asterisks indicate levels of significance (**p* < 0.05; ***p* < 0.01; *** *p* < 0.001). APs, aquaculture ponds; MFs, mudflats; SA, *S. alterniflora* marshes; MFs-SAs, the transition from MFs to SAs; SAs-APs, the transition from SAs to APs.

**Fig. S3** Assembly processes of protistan communities assessed by Jaccard index (a) and Bray-Curtis index (b) based on Modified Stochasticity Ratio (MST) in the three wetland habitat types. MST < 0.5 denotes more deterministic assembly while MST > 0.5 denotes more stochastic assembly. Asterisks indicate levels of significance (NS *p* ≥ 0.05; * *p* < 0.05; ** *p* < 0.01; *** *p* < 0.001). APs, aquaculture ponds; MFs, mudflats; SA, *S. alterniflora* marshes.

Fig. S1


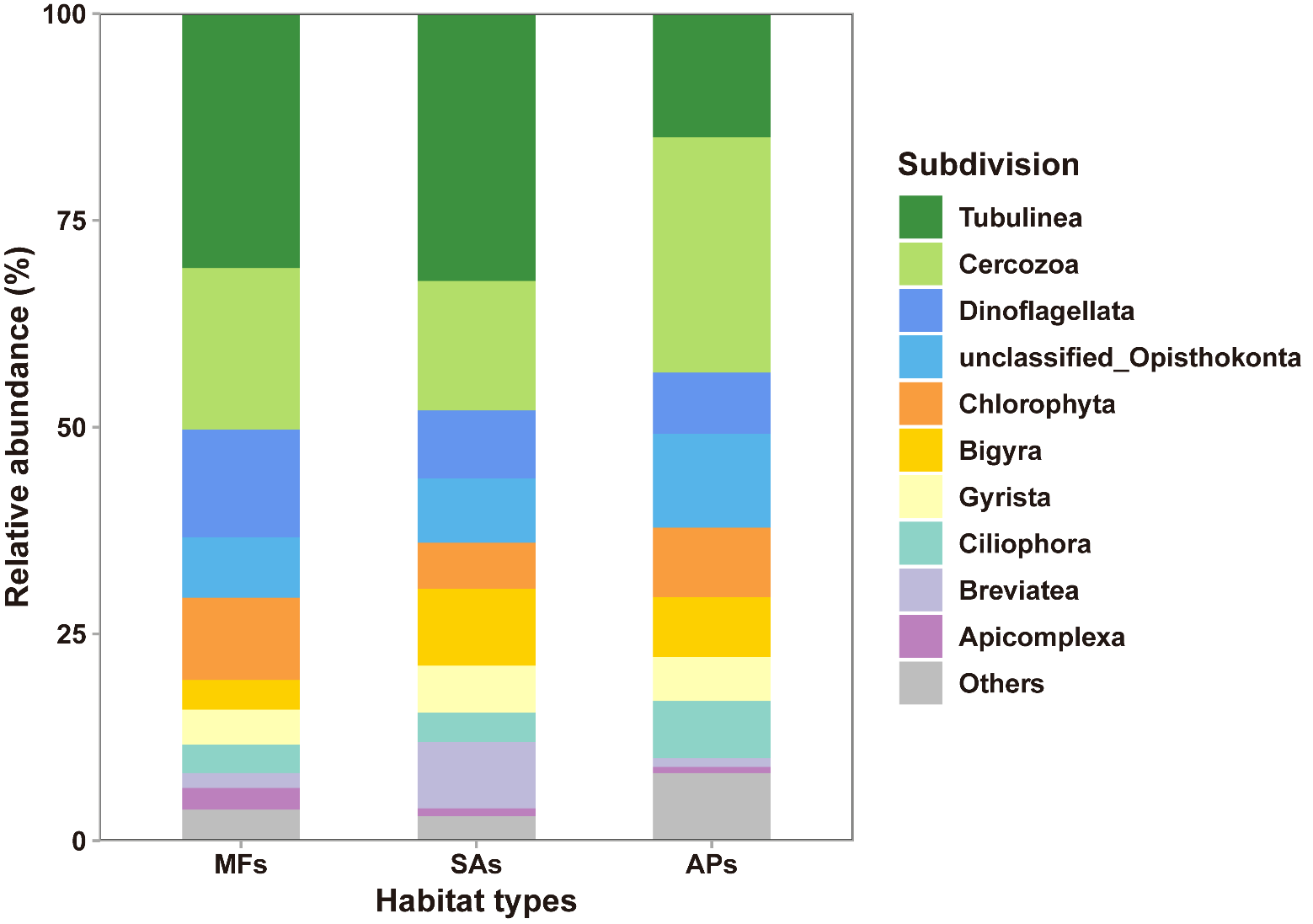


.

Fig. S2


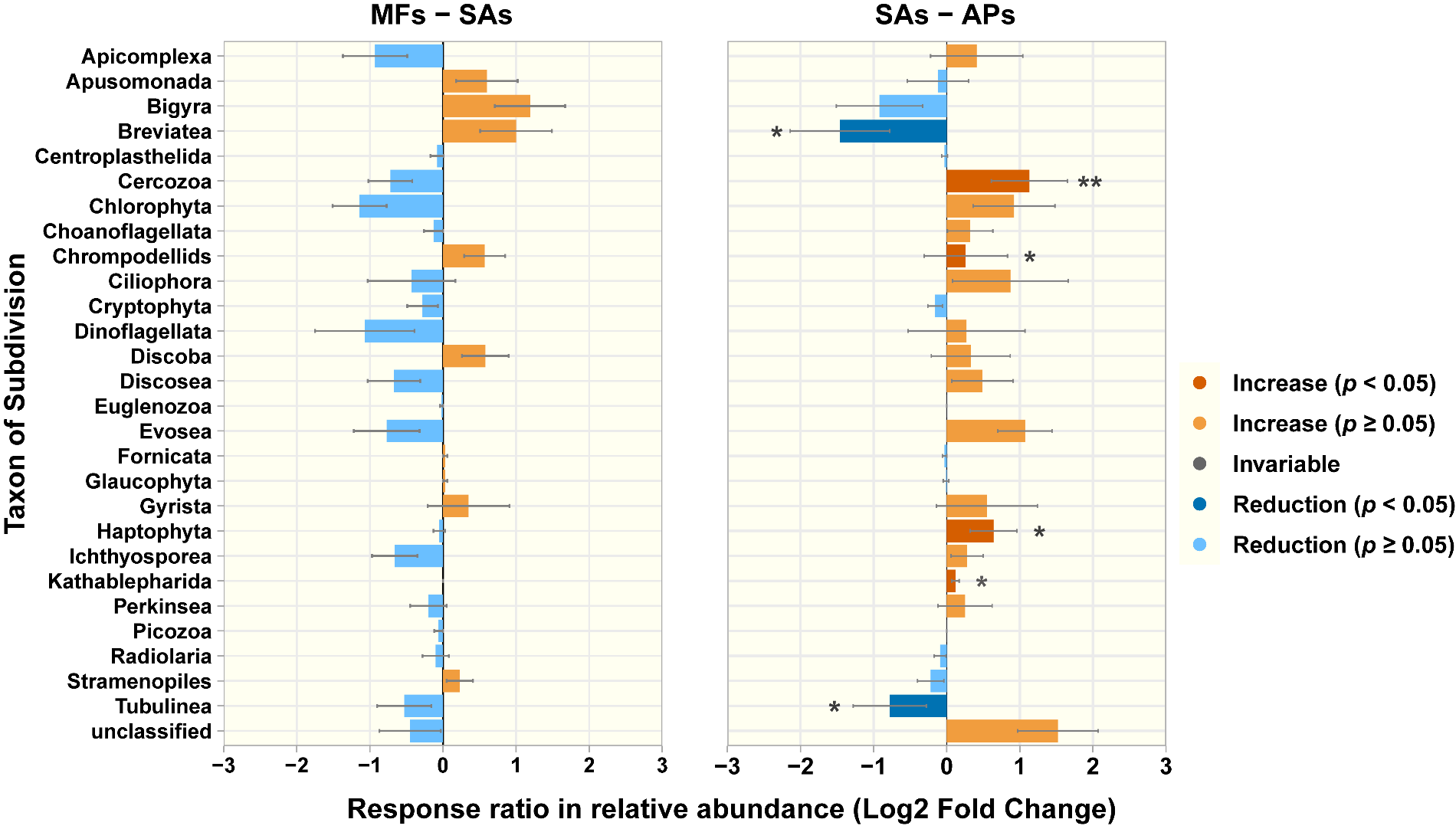


Fig. S3


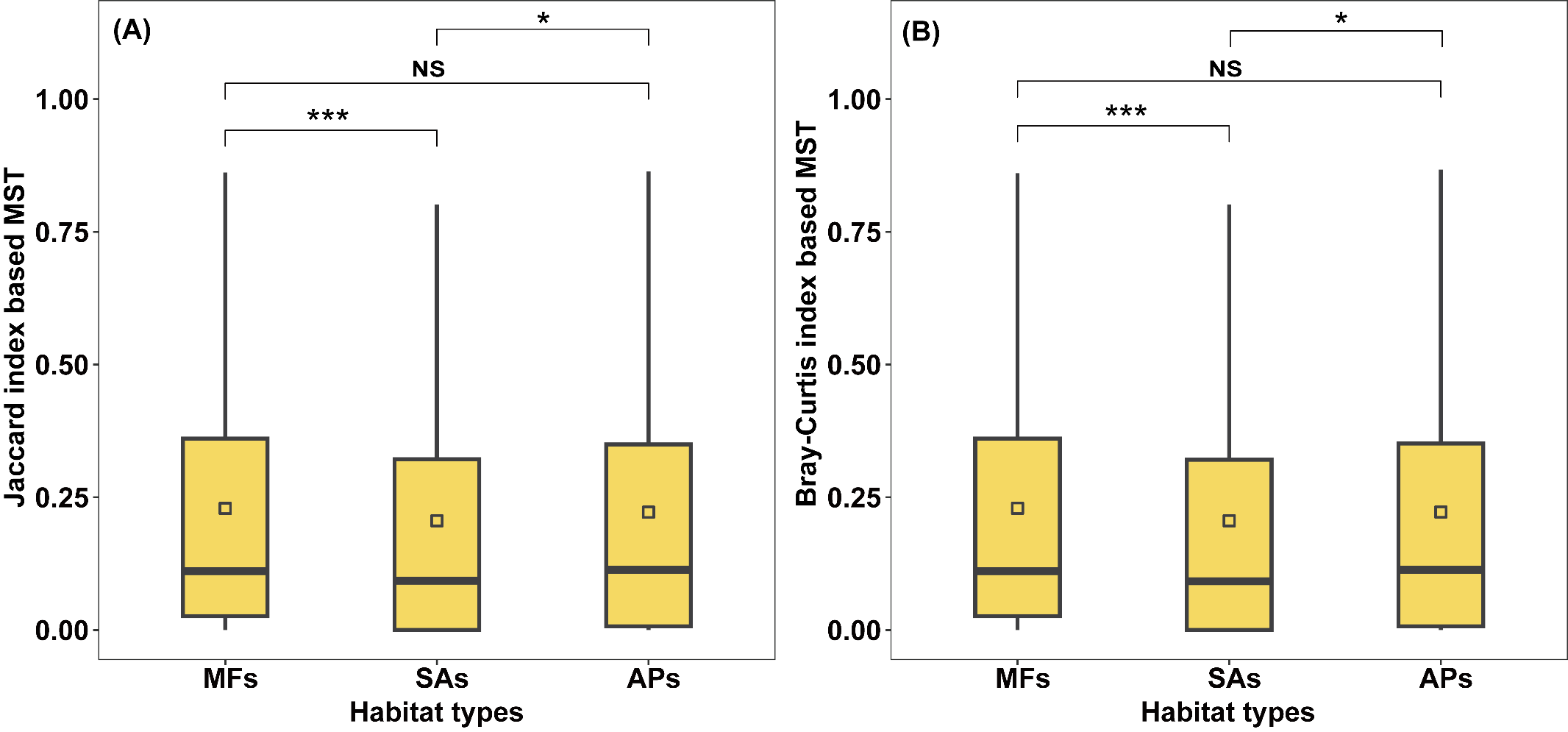

Supplement: Supplemental material — Table S1; Figures S1 to S3. [file aem.01661-25-s0001.docx]
